# Challenges Posed by Immune Responses to AAV Vectors: Addressing Root Causes

**DOI:** 10.3389/fimmu.2021.675897

**Published:** 2021-05-17

**Authors:** Bradley A. Hamilton, J. Fraser Wright

**Affiliations:** Center for Definitive and Curative Medicine, Division of Hematology, Oncology, Stem Cell Transplantation and Regenerative Medicine, Department of Pediatrics, Stanford University School of Medicine, Stanford, CA, United States

**Keywords:** AAV, gene therapy, immunogenicity, PAMPS, TLR9, CpG

## Abstract

Host immune responses that limit durable therapeutic gene expression and cause clinically significant inflammation remain a major barrier to broadly successful development of adeno-associated virus (AAV)-based human gene therapies. In this article, mechanisms of humoral and cellular immune responses to the viral vector are discussed. A perspective is provided that removal of pathogen-associated molecular patterns in AAV vector genomes to prevent the generation of innate immune danger signals following administration is a key strategy to overcome immunological barriers.

## Introduction

Human gene therapies using recombinant adeno-associated virus (rAAV) vectors have demonstrated enormous promise. While rAAVs lack many characteristics of the wild-type viruses from which they are derived, they nevertheless retain legacy immunogenic features that affect their safety and efficacy. Marked inflammatory toxicities that have recently been observed as vector dosing has increased, including complement activation, cytopenias and severe hepatotoxicity (1), likely represent part of the continuum of diverse aspects of clinical immune responses that have emerged over the last two decades. Accurate identification and removal where possible of the immunogenic features in the vector design process, in conjunction with the optimization of immunomodulation protocols, will minimize rAAV-associated immunotoxicities and enhance therapeutic benefit. Process and product-related impurities are known to contribute to unwanted immune responses in biologics generally, and even highly purified rAAV vectors contain unique product-related impurities that present immunogenic risks (2). However, rAAV vectors comprising the viral capsid proteins (VP1, 2, 3), the vector DNA expression cassette, and the products generated by its transcription and translation are themselves immunogenic. Several comprehensive reviews of host immune responses to AAV gene therapy have been recently published (3–7). This article focuses on the inherent immunogenic features of rAAV vectors and provides a perspective that the removal of pathogen associated molecular patterns (PAMPs) from vector genomes is key to preventing pre-requisite innate immune signals that lead to deleterious adaptive immune responses.

**Figure 1:**
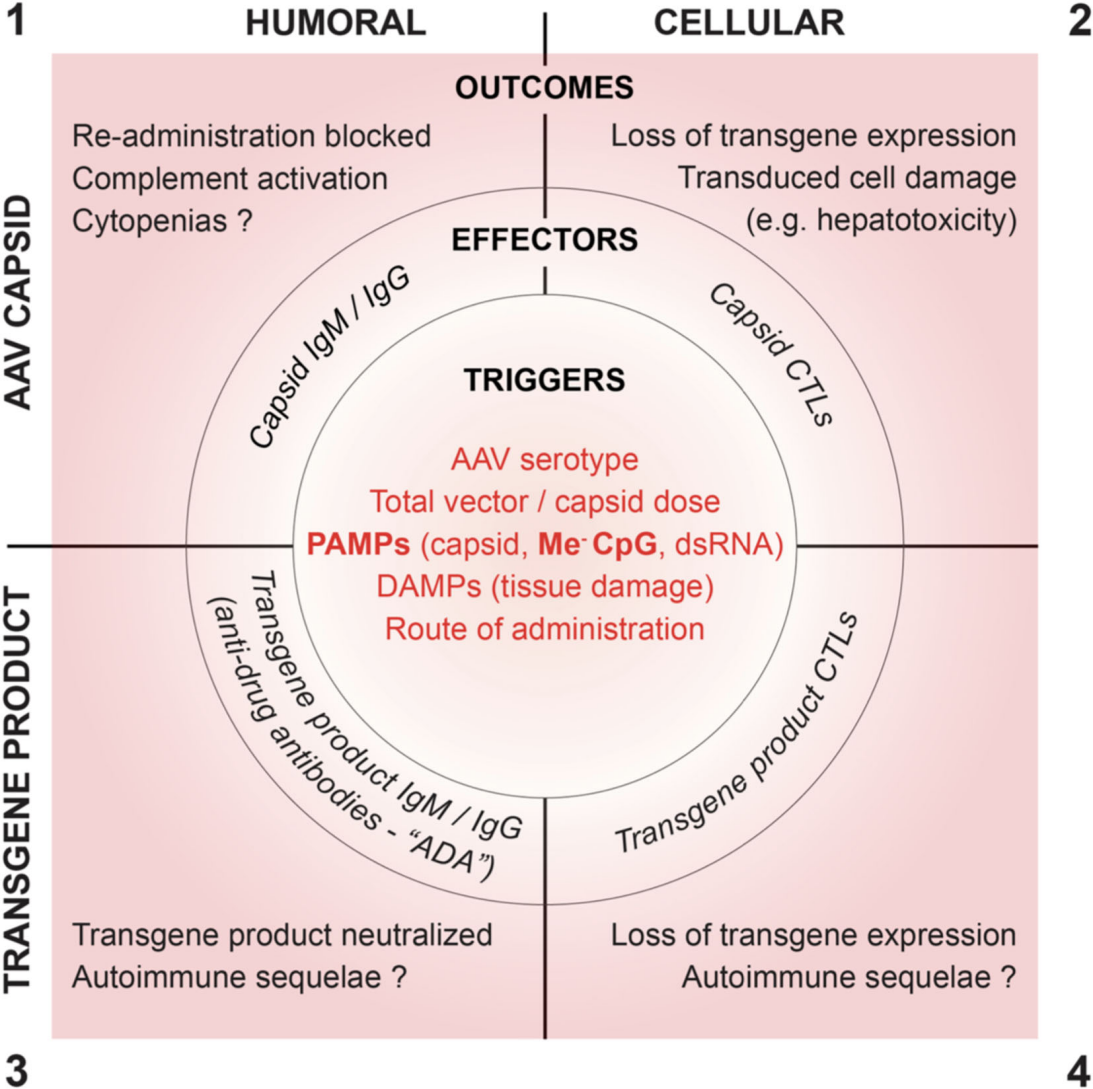
Proposed root cause triggers, effector functions and clinical outcomes of humoral and cellular immune responses to AAV vectors.

Recombinant AAV capsids and their transgene products can provide targets for humoral and cellular immunity. Conceptually, immune responses to rAAV can be categorized into the quadrants shown in **Figure 1**, which summarizes innate immune triggers, adaptive effector functions, and clinical sequelae.

### Innate Immune Signals as Key Triggers for Deleterious Immune Responses

Robust immune responses require three signals (8). A pre-requisite innate immune danger signal (Signal 0) generated by binding of pathogen-associated and/or danger-associated molecular patterns (PAMPs and DAMPs) to host pattern recognition receptors (PRRs) results in pro-inflammatory cytokine release. An antigen-specific signal (Signal 1), generated by binding of non-self antigens or peptide/MHC complexes to antigen-specific receptors on B or T cells, respectively, then leads to clonal expansion of antigen-specific effector antibodies and cytotoxic T-lymphocytes (CTLs). Co-stimulatory signals (Signal 2), generated by additional receptor/ligand interactions between antigen-specific effector lymphocytes and helper T cells or professional antigen presenting cells, amplify effector functions and prevent anergy. Preventing generation of the initial danger signals by removal of PAMPs is a root cause-focused strategy to prevent immune responses to rAAV vectors. Three rAAV-associated PAMPs have been described: i) ligands present on rAAV viral capsids that bind toll-like receptor 2 (TLR2), a cell-surface PRR on non-parenchymal cells in the liver (9); ii) unmethylated CpG dinucleotides in viral DNA that bind TLR9, an endosomal PRR in plasmacytoid dendritic cells (pDCs) and B cells (10, 11); and iii) double-stranded RNA formed as a result of the bi-directional promoter activity associated with AAV inverted terminal repeats (ITRs), which binds melanoma differentiation-associated protein 5 (MDA5), a cytoplasmic PRR (12). Removal of these PAMPs by vector design optimization to avoid generation of the initial danger signal is predicted to markedly reduce immune responses to AAV.

## Humoral Immune Responses to AAV

### AAV Capsid Antibodies

The generation of antibodies to the rAAV capsid is an expected outcome following challenge of the immune response with a non-self-i.e., viral protein, and generation of high titer capsid antibodies has been consistently reported following vector administration to human subjects. Tissue macrophages take up rAAV non-specifically by phagocytosis, process the capsid, and following migration to a draining lymph node, present capsid-derived peptides to effector lymphocytes. The presentation of AAV peptides on MHC class II molecules, with co-stimulatory signals, activates capsid-specific CD4+ helper T cells and B cells (10). T_h_2 cells produce interleukins that stimulate B cell proliferation and antibody class switching, leading to increased neutralizing antibody production. A robust humoral immune response requires engagement of T_FH_ cell CD154 with CD40 on B cells. After germinal center formation, B cells differentiate into affinity matured plasma cells, antibody class switching from IgM to IgG occurs, and B memory cells are generated (13).

Prior exposure to wild-type AAV by natural infection or to rAAV by prior *in vivo* gene therapy results in neutralizing antibodies in serum that efficiently block target cell transduction and thereby therapeutic efficacy of systemically administered rAAV (14, 15). Potential participants are excluded from rAAV clinical trials if significant titers of neutralizing antibodies are measured in their sera during screening. Natural infections are common, and 70% of human sera contain antibodies against rAAV1 and rAAV2, 45% against rAAV6 and rAAV9, and 38% against rAAV8 (16). Children as young as two years of age are often seroconverted, with antibodies against multiple AAV serotypes (17), and antibodies have been shown to cross react between rAAV serotypes (18). Administration of rAAV to seronegative individuals resulting in high titer antibody formation will similarly block the option of systemic re-administration of AAV-based gene therapy vectors for many years. Capsid antibodies can also contribute to inflammation and cytotoxicity by activating the classical pathway of complement, and by mediating uptake by FcR-expressing immune cells such as dendritic cells and macrophages, potentiating AAV specific adaptive immune response and generation of proinflammatory cytokines (19).

### Overcoming Challenges Posed by AAV Capsid Antibodies

To facilitate rAAV-mediated gene therapy in a broader patient population, several strategies are being developed to circumvent the barrier posed by capsid antibodies. Capsid modification by rational design or screening of capsid variant libraries to identify those that avoid recognition by AAV antibodies prevalent in human sera are two approaches (20). Novel capsids identified by modification of AAV2 following antibody epitope mapping and targeted disruption of epitopes by mutagenesis were reported to reduce neutralization by high titer individual human sera up to 42-fold and by pooled human serum IgG (IVIG) up to 10-fold (21). Developing AAV capsids in which the epitopes to prevalent human antibodies have been eliminated is an important strategy, but will not enable vector re-administration if needed due to waning or loss of expression following a first administration. If feasible, the development of stealth vectors with surface viral epitopes masked by non-antigenic features that do not interfere with target cell transduction (20) would be transformative to enable AAV administration in the presence of capsid antibodies. Alternative approaches to transiently remove antibodies in subjects prior to vector administration include *in vivo* enzymatic digestion to degrade total IgG (22) and plasmapheresis (23, 24) to remove total (23) or capsid-specific (24) IgG. Maneuvers to deplete antibodies present risks that need to be balanced with the potential benefits of gene transfer.

### Complement Activation

Complement activation following high dose systemic administration is a recently described inflammatory toxicity associated with high dose rAAV administration (1). The complement system is comprised of over 30 proteins mainly synthesized in the liver and circulating in serum and interstitial fluid (25) that collectively play an important role in the recognition and elimination of pathogens (25). Complement acts by opsonizing pathogens and antigens for phagocytosis, co-stimulating B cell activation and antibody production, and by forming a membrane attack complex (MAC) for direct killing, among other functions. Complement can be triggered by the lectin pathway, the classical pathway, and the alternative pathway. The classical pathway is initiated when the antibody Fc regions of immune complexes composed of antigen-bound IgM or IgG bind complement component C1 causing confirmational changes activating C3 convertase, which cleaves C3 into fragments C3a and C3b. Soluble C3a recruits macrophages and neutrophils to the site of infection, and TNFα, IL-1, and IL-6 secretion by these leukocytes amplify the production of complement proteins (25). Membrane-bound C3b opsonizes antigens to facilitate their removal and propagates the formation of the MAC. Immune complexes composed of rAAV bound by IgM or IgG, formed when high doses of rAAV vector are administered to individuals with pre-existing or rapidly formed capsid antibodies, if deposited within recipient tissues would be predicted to cause complement activation-associated host cell damage. Covalent deposition of complement component C3b on AAV particles mediated uptake of the vector in a human monocyte cell line leading to production of pro-inflammatory cytokines (26). This highlights that AAV immune complexes involving pre-existing or newly formed capsid antibodies have the potential to activate complement and amplify capsid immune responses after AAV-mediated *in vivo* human gene transfer. Complement inhibitors are being investigated for their ability to modulate immune responses to AAV gene therapy vectors, including APL-9 as a C3 inhibitor, and Eculizumab as a C5 inhibitor (27).

## Cellular Immune Responses to AAV

### Cytotoxic T Lymphocytes

As a central feature of immune responses to viruses, the generation of CD8+ capsid specific cytotoxic T lymphocytes (CTLs) is an expected outcome following infection with wild type AAV. Capsid CTLs were not anticipated in early clinical trials using rAAV because vectors do not encode viral antigens in their genomes. However, capsid CTLs have been frequently reported following rAAV vector administration in humans and associated with the elimination of transduced cells. Both transduced target cells and professional antigen presenting cells (APCs) present capsid-derived peptide epitopes on MHC class 1 molecules to CD8+ T cells (28–30). After activation and expansion, the resulting CTLs have the potential to destroy rAAV-transduced cells, inducing inflammation in the target organ and adversely affecting the gene transfer outcome (14, 31). Herzog’s group (29, 32), described the dendritic cell-dependent mechanism of CD8+T cell response against the AAV capsid. After endocytosis and partial degradation of rAAV particles in their lysosomal compartment, plasmacytoid dendritic cells (pDCs) present in lymph nodes or the spleen will generate type 1 interferons as a danger signal if CpG PAMP DNA is detected by TLR9. Conventional dendritic cells (cDCs) in the vicinity that have also taken up rAAV particles present capsid peptides on MHC class 2 molecules to CD4+ helper T cells, resulting in the generation of an antigen specific signal. These signals together promote maturation of the cDC, presentation of capsid peptides by MHC class 1 molecules to and activation of capsid-specific CD8+ CTLs that can then migrate to other tissues and eliminate transduced cells.

### Systemic AAV Administration: Experience in Hemophilia Clinical Trials

In the first hemophilia B gene therapy clinical trial that reported therapeutic but transient levels of the FIX transgene product, elimination of vector-transduced hepatocytes by capsid-specific CTLs began four weeks after vector administration (14). Since that initial trial, several additional hemophilia B gene therapy trials have been reported, some achieving long-term efficacy, but others recapitulating the loss of transgene expression associated with immune-mediated loss of transduced cells (33). This failed outcome was recently reported in a trial using an AAV8 vector (34), demonstrating that consensus strategies to effectively minimize and manage deleterious capsid CTL response have not yet been fully established. The targeted nature of these efficacy-limiting responses when they are observed is evidenced by liver enzyme elevation concomitant with loss of therapeutic transgene expression. The timeframe of CTL effector manifestation after rAAV administration is delayed compared the kinetics following wild-type virus infection. One explanation is that, in contrast to the direct processing for MHC class I presentation of viral antigens following their transcription and translation during natural virus infections, the capsid protein component of the rAAV dose must be processed *via* the indirect pathway (14, 28), which is less efficient. Furthermore, the high stability of AAV capsids would be predicted to contribute to slow capsid processing by the indirect pathway (35). Alternatively, a delayed innate signal from dsRNA generated following vector transduction has been proposed to contribute to the delayed formation of capsid CTLs (12). In any case, the vector dose inoculum as the sole source of capsid antigen predicts that peptide presentation by MHC class I on transduced cells will be transient.

### Intramuscular Administration: Experience in AAT-Deficiency Clinical Trials

Brantly and colleagues reported the occurrence of cellular immune responses in a clinical trial for alpha-1-antitrypsin (AAT) deficiency, including IFN-gamma ELISPOT responses to AAV capsid, that did not completely eliminate transduced cells (36). Capsid-specific T cells were detected at day 14 and remained present at day 90, yet subjects in the high dose cohort demonstrated sustained expression of AAT. Biopsy samples taken at three months post vector administration demonstrated inflammatory cells that were still present, although at a lower level at 1 year. Phenotyping revealed a substantial portion of the T cells present were regulatory T cells (Tregs), supporting that following intramuscular administration, rAAV induces Treg responses that allow ongoing transgene expression (37). The development of Tregs that provide immune tolerance to the therapeutic transgene product has also been shown to be induced by gene transfer to the liver by AAV (38), supporting that some level of liver transduction even when the primary target for gene expression is a different tissue may be beneficial (39).

### Overcoming Immune Responses by Immune-Suppression

The inhibition of both capsid peptide presentation by MHC class I molecules and expansion of capsid-specific CTLs, each required for transduced cell lysis, have been proposed or used to prevent the elimination of vector-transduced cells and thereby enable long-term transgene expression. Proteosome inhibitors have been shown to prevent capsid processing by the indirect pathway (40, 41). Transient immune suppression with prednisone was used to block CTLs in subjects that experienced elevated liver enzymes in the first AAV gene therapy trial that reported therapeutic and durable expression of FIX (42). Prednisone initiated prior to vector administration and continued for at least 30 days is part of the administration protocol for Zolgensma, the first licensed AAV product approved for systemic administration, and similar protocols are used for many AAV products currently in clinical development. While prophylactic corticosteroids have improved outcomes in AAV clinical trials, they have failed to prevent loss of transgene expression in others (34), and present risks (34, 43). Additional novel immunomodulatory approaches have shown promise. The co-administration of polylactic acid nanoparticles containing rapamycin (sirolimus) can tolerize the non-human primate (NHP) immune system to the transgene product (44). However, blocking the interleukin-2 receptor with daclizumab to inhibit effector T cell activation in NHPs increased immunity against the transgene (45). Preclinical studies of rituximab-mediated B cell depletion (46), and mycophenolate mofetil depletion of guanosine nucleotides to arrest T cells and B cells (45), prevented anti-capsid humoral and cell-mediated responses. The standardization of clinical immunomodulatory protocols has been complicated by the absence of an animal model in which vector immunogenicity can be reliably studied and predicted. The risks presented by immunomodulation, such as nosocomial and opportunistic infections, must be weighed against the potential benefits of gene therapy for each disease indication.

### Overcoming Immune Responses by Improved Vector Design

Vector dose, serotype, genome configuration and method of manufacture have all been proposed as important factors contributing to prednisone-resistant loss of transgene expression. However, none of these factors consistently correlate with clinical observations, suggesting a different vector attribute(s) is key. Compelling evidence for a key role of unmethylated CpG motifs in efficacy-limiting immunotoxicity in AAV clinical trials has emerged. The recognition of unmethylated CpG motifs by TLR9 leads to its dimerization and downstream activation *via* MyD88 in professional APCs (11, 47, 48). The frequency of CpG dinucleotides in most microbial genomes approximates 6.25%, the frequency based on random nucleotide utilization, and most microorganisms do not methylate CpG dinucleotides. In contrast, the 0.97% frequency of CpGs in the human genome is markedly suppressed, and CpG dinucleotides in human DNA are predominantly methylated so that the frequency of unmethylated CpGs in human DNA is > 20-fold lower than in bacterial DNA. This difference provides the basis for discrimination between human and microbial DNA by TLR9. AAV expression cassettes that include components derived from viral or microbial sources such as AAV ITRs, viral promoters, and enhancers such as WPRE will therefore contain PAMP CpG motifs from these sources.

Increasing the number of CpG motifs in plasmid DNA vaccines is an effective way to increase both cellular and humoral immune responses (49–51), and plasmid methylation markedly reduces those responses (50, 51). These findings establish a key role for unmethylated CpG-triggered TLR9-MyD88 pathway activation in strong cellular and humoral immune responses. The well-established role of unmethylated CpG motifs as adjuvants in DNA vaccines further highlights the need, by vector genome and production process design, to reduce the frequency of unmethylated CpGs in AAV vector genomes to a level below the threshold that activates human TLR9.

Innate immunostimulatory CpG motifs (CpG PAMPs) are unexpectedly abundant in AAV vectors because of their hypomethylation. Using BrdU labeling to assess the origin of rAAV genomes, Hauck and colleagues reported that a large percentage of AAV2 vectors generated by transient transfection of HEK293 cells contain expression cassettes rescued directly from plasmid DNA (52). The authors proposed that the consequent high frequency of unmethylated CpG dinucleotides in AAV vector genomes contributed to clinical efficacy-limiting immune responses. The hypomethylation of CpGs in AAV vector genomes was further characterized by direct biochemical analysis (53, 54) supporting that, in addition to vector genome rescue from plasmid DNA, the kinetics of vector genome packaging leads to low methylation even in mammalian production cells that would normally achieve human levels of CpG methylation. CpG hypomethylation is a non-human epigenetic feature that explains why even wild-type human DNA sequences that do not bind TLR9 when they are predominantly methylated i.e., in the human genome, become TLR9-activating CpG PAMPs in AAV vector genomes.

Validation of the deleterious effect of CpG motifs in AAV vector genomes has been clearly demonstrated in animal models (10, 55–57), including demonstration that CpG-depleted AAV vectors evade immune responses (56), and human clinical trials (33, 34). The use of higher CpG content vectors correlated with hepatoxicity and absence of durable transgene expression in multiple hemophilia B gene therapy trials (33).

Synonymous codon substitutions to replace native CpG dinucleotides, and CpG methylation to ‘humanize’ the AAV genome are two approaches to remove TLR9-recognized PAMPs in AAV expression cassettes. The reduction of CpG dinucleotides within AAV vector genomes, especially those within motifs known to be strong activators of the TLR9 pathway, is supported as a key practical strategy to reduce adaptive immune response to AAV gene therapy vectors. Concerns with synonymous codon substitutions include deviations from naturally evolved codon usage patterns that may result in transgene product misfolding (58, 59). Components of the AAV genomes outside of the ORF, such as ITRs and promoters, are equally ‘visible’ to TLR9 receptors, and are often rich in CpG dinucleotides. **Table 1** lists calculated TLR9 activation risk factors for non-ORF components of AAV vector expression cassettes. If achievable, methylation of CpG motifs to ‘humanize’ this epigenic attribute is predicted to reduce TLR9 activation. Another approach is the incorporation of TLR9 inhibitory nucleotides sequences into the AAV expression cassette (60).

**Table 1:** TLR9 Activation Potential and Risk Factor (RF)* calculations for AAV genomes and components.

DNA test article	Size (nt)	RF_1_	RF_3_	Normalized RF_3_	TLR9 Activation
*Human genome*					
complete genome ^*^	3.21 x10^9^	0.965	0.191	**1.00**	**-**
*Bacterial genome*					
Escherichia coli ^*^	4.64 x10^6^	7.471	4.683	24.5	**+++**
*Clinical rAAV genomes*					
low CpG AAV8-FIXsc (scAAV2/8-LP1-hFIXco^42^; clinicaltrials.gov NCT00979238)	4611	1.757	1.298	6.80	**+**
high CpG AAV8-FIXsc (BAX335^34^; clinicaltrials.gov NCT01687608)	4780	5.859	4.383	22.9	**+++**
*rAAV genome components*					
AAV2 ITRs	145	11.03	7.862	41.2	**+++**
ApoE HCR hAAT Promoter	828	3.019	2.754	14.4	**+++**
MHCK7 Promoter	770	2.078	1.851	9.69	**++**
CAG Promoter	584	8.562	2.603	13.6	**+++**
CMV Promoter	508	5.709	3.740	19.6	**+++**
WPRE	597	6.198	3.819	20.0	**+++**

*Wright JF. Quantification of CpG motifs in rAAV genomes: avoiding the toll. Mol Ther 2020; **28**(8):1756-58.

RF_1_ = f [CpG_T_ / nt] X 100%.

RF_3_ = f [CpG_T_ + CpG_S4_ – 2CpG_I4_ / nt] X f [CpGMe^neg^ / CpG_T_ ] X 100%.

Normalized RF_3_ = RF_3_ (test article) / RF_3_ (human genome).

## The Patient Perspective

The importance of effectively reducing the immunogenicity and potential for inflammatory toxicities during AAV-mediated gene therapies is emphasized by patient considerations. The weight participants bear in participating in rAAV clinical trials is compounded by the fact that failure to achieve efficacy after one administration is likely to preclude benefit from future improved AAV vectors. This long-term adverse outcome emphasizes the moral imperative towards scientific communication and data sharing to validate the vector attributes involved and to develop effective management of AAV immune responses towards the goals of low immunotoxicity and durable therapeutic transgene expression.

## Conclusion

In order for potentially curative and definitive AAV-based gene therapies to be applicable to a broader range of indications and reach more patients, immune responses to rAAV must be reduced and better controlled. The formation of anti-capsid antibodies restricts rAAV gene therapy to a single administration at least for the commonly used systemic route of administration. Therefore, trials that seroconvert human subjects without a reasonable expectation of clinical benefit, in particular those using vectors containing known significant innate immunogenic features, should not be performed. Furthermore, while capsid-specific CTL responses can sometimes be controlled by immune suppression, many diseases necessitate systemic administration of rAAV at high doses where immunomodulation is less effective, and inflammatory toxicities such as complement activation are likely to be worsened by strong innate immunogenic features of the vector. An effective strategy must combine immune-modulation with better design of vectors – ‘humanized vectors’ with reduced potential to trigger efficacy-limiting immune responses. The role of TLR9 activation as a seminal potentiator of cellular and humoral immune responses to rAAV is under-appreciated, and reducing TLR9 signaling by reducing the frequency of unmethylated CpG motifs in vector genomes is an important key to realizing the enormous promise of rAAV mediated gene therapy.

## Data Availability Statement

The raw data supporting the conclusions of this article will be made available by the authors, without undue reservation.

## Author Contributions

All authors listed have made a substantial, direct, and intellectual contribution to the work and approved it for publication.

## Conflict of Interest

JFW is a co-founder of Spark Therapeutics and Kriya Therapeutics, consults to companies developing rAAV-based gene therapy products, and is inventor of patents relating to recombinant viral vector design and manufacture.

BAH declares that the research was conducted in the absence of any commercial or financial relationships that could be construed as a potential conflict of interest.
